# Live biotherapeutic products: a capstone for prevention of recurrent *Clostridiodes difficile* infection

**DOI:** 10.3389/frmbi.2024.1399440

**Published:** 2024-05-16

**Authors:** Kanika Sehgal, Paul Feuerstadt

**Affiliations:** ^1^ Department of Internal Medicine, Yale School of Medicine, New Haven, CT, United States; ^2^ Division of Digestive Diseases, Yale School of Medicine, New Haven, CT, United States; ^3^ Department of Gastroenterology, Physicians Alliance of Connecticut (PACT) Gastroenterology Center, Hamden, CT, United States

**Keywords:** *Clostridioides (Clostridium) difficile* infection, *difficile*, fecal micriobiota transplantation, FMT, live biotherapeutic product

## Abstract

*Clostridiodes difficile* infection (CDI) continues to be one of the leading causes of healthcare-acquired diarrhea and infections, and recurrence is the biggest challenge in its management. As technology and research have led to a better understanding of the pathophysiology of *C. difficile*, we have come to appreciate the role that the gastrointestinal microbiota plays in infection onset and the prevention of recurrence. The gut microbiota is disrupted in those with CDI, which allows further propagation of the infection leading to recurrence, if the microbiota deficiency is unable to regrow itself. While antimicrobial therapy is necessary for treatment of any CDI, these therapeutics do not address the underlying disturbance of microbiota. Microbial remodulation therapies have been developed supplementing the microbiota deficiency that exists after the standard of care antimicrobial resulting in a reduction of recurrence. Fecal microbiota transplantation (FMT) was the initial attempt for this type of therapeutic and proved to be safe and effective, however never achieved FDA approval. In light of this, live biotherapeutic products (LBPs) were developed by pharmaceutical companies through a more standardized and regulated process. These products are safe and efficacious in reducing CDI recurrence when given after a standard of care antimicrobial, eventually leading to FDA approval of two products that can now be used widely in clinical practice.

## 1 Introduction

*Clostridioides difficile* infection (CDI) continues to be a leading cause of antibiotic associated and healthcare related diarrhea in the United States (Lessa et al., 2015; Magill et al., 2018). With an estimated half million annual infections, approximately 30,000 deaths in the United States and a crude overall incidence rate of 121.2 cases per 100,000 persons, the burden of CDI is tremendous (Lessa et al., 2015; Centers for Disease Control and Prevention (CDC), 2019; Guh et al., 2020). One of the biggest challenges in managing CDI is recurrence. After an initial episode, recurrence rates have been estimated to be as high as 35%, and this rate increases with each successive episode, including up to 45% after the first recurrence and ~60% after the second recurrence (McFarland et al., 2002; Pepin et al., 2005; McDonald et al., 2018).

The treatment of CDI has historically included antimicrobials as a singular therapy. The American College of Gastroenterology guidelines published in 2021 recommend the use of vancomycin, fidaxomicin or metronidazole for non-severe initial infection (Kelly et al., 2021a). For severe initial infection (defined as a white count of >15,000 cells/mm^3^ and/or serum creatinine >1.5 mg/dL) treatment with either vancomycin or fidaxomicin is recommended. For an initial recurrence, a taper-pulse regimen of vancomycin or a course of fidaxomicin should be considered. As technology and research have led to a better understanding of the pathophysiology of CDI, we have come to appreciate the role of the microbiota in preventing recurrence and with this, additional therapeutics have been developed supplementing the standard of care (SOC) antimicrobial resulting in a reduction of rates of recurrence (Seekatz and Young, 2014).

The vegetative phase of *C. difficile* releases toxins causing symptoms including abdominal pains, fevers and diarrhea. Standard of care antimicrobials, such as vancomycin and fidaxomicin, control the vegetative phase of the infection. The spore phase is more resilient transferring from patient to patient, and also remaining within a patient’s system after SOC antimicrobials for extended periods. Antimicrobials have little to no impact on the spore phase, however, a healthy diverse microbiota can eradicate this phase decreasing the likelihood that it reconverts, or germinates, back into the vegetative phase, causing a recurrence (Seekatz and Young, 2014).

With this in mind, the treatment landscape for recurrent CDI (rCDI) has evolved dramatically in the last ten to fifteen years. A disruption of the gut microbiome composition, known as dysbiosis, allows spore germination, reduces the inherent resistance against colonization *with C. difficile* allowing vegetative growth with toxin production (Seekatz et al., 2016). Prior studies have demonstrated that low microbial diversity, particularly a decrease of the bacterial phyla *Bacteroidetes* and *Firmicutes*, plays a vital role in the pathogenesis of CDI (Chang et al., 2008). Thus, while antimicrobial therapy is necessary for treatment of CDI, these treatment modalities cannot directly address or correct the underlying dysbiosis. With SOC antimicrobials alone, we rely on natural regrowth of the deficient *Bacteroidetes* and *Firmicutes*, which frequently does not occur appropriately resulting in the high rates of recurrence of CDI. In fact, vancomycin itself can further deplete the diversity of the microbiota leaving patients prone to getting CDI recurrence (Louie et al., 2009; Ajami et al., 2018). In the light of this, therapies targeting intestinal microbial restoration following antimicrobials, thereby treating both phases of the infection, have rapidly evolved and are now widely available.

Therapies replenishing the gut microbial diversity are designed to engraft desirable microorganisms that are deficient, regaining the so called “colonization resistance.” This was originally achieved via fecal microbiota transplantation (FMT) but in recent times, the more sophisticated approach of live biotherapeutic products (LBPs) have become available. Broadly, FMT involves the transfer of stool from a healthy screened donor into the intestinal tract of the recipient, whereas the United States Food and Drug Administration (FDA) approved LBPs widely screen donors, sample the stool for consistency of microbes to be transferred and have standardized approaches for administration. This pivotal change in management underlies the progress achieved within the therapeutics for rCDI. This manuscript will outline the differences between thes two FDA approved LBPs, and clinical trial evidence related to the efficacy of LBPs.

## 2 Fecal microbiota transplantation

The principle of FMT, as it relates to CDI, is to replenish the entire microbial diversity supplementing the deficiencies of desirable bacteria within the intestinal tract, particularly *Bacteroidetes* and *Firmicutes.* This introduction of a broad array of microbes aims to reverse the dysbiosis created by CDI establishing metabolic equilibrium that minimizes the likelihood of future recurrent episodes. Guidelines from the Infectious Disease Society of America and Society of Healthcare Epidemiology of America in 2021, along with the American College of Gastroenterology 2021, recommend the use of FMT after two or more recurrences of CDI that have been treated with appropriate antibiotics (Johnson et al., 2021; Kelly et al., 2021a). The American Gastroenterological Association guideline discussing fecal microbiota-based therapies published in 2024 advised consideration for fecal-microbiota based therapies, such as FMT and LBPs, in adults with “recurrent *C. difficile”* (Peery et al., 2024). This effectively includes patients with first recurrence, expanding the indications for these treatments.

Although the clinical trials considering FMT are heterogenous in reporting of methodology and design there are now many studies considering FMT for the prevention of recurrence of CDI (Feuerstadt et al., 2022a). The efficacy for FMT to prevent rCDI has been seen to vary, being more than 85% in many early observational studies but closer to 72% in controlled clinical trials (McGovern et al., 2021). An observational study of the largest stool bank in the United States that utilized centralized screening and processing, assessed 5,344 patients between 2014 and 2018 reporting a clinical resolution rate of 78% (Osman et al., 2022). Another study reported prospectively collected data from the AGA FMT National Registry, reporting that across 259 patients, they had 1 month follow up data on 222 with clinical resolution rates of 90% at that time. Of the 112 patients that had data available for 6-month follow up, 96.4% remained without recurrence (Kelly et al., 2021b).

A recent systematic review and meta-analysis evaluated the efficacy of FMT and LBPs for the prevention of rCDI in prospective clinical trials and compared this to the data from randomized clinical trials (Tariq et al., 2023). Across 19 clinical trials, clinical resolution with FMT or LBP was seen in 78% of patients, and among these, a resolution rate of 72% was observed in the 10 trials that included a control arm. While data from most studies have been encouraging, there has been significant heterogeneity in study design, screening of donors, composition of microbiota that was administered, assessment of outcomes and mode of administration among these clinical trials. Given this lack of standardization across studies with FMT, efforts focusing on microbiota therapeutics that are standardized with consistent microbial consortia have created the sub-category of LBPs (**Table 1**). This advancement of technology has resulted in better studies and resulted in the FDA approval of two products that will be discussed below.

**Table 1:** Key features of difference between fecal microbiota transplantation and live biotherapeutic products.

		Fecal Microbiota Transplantation	Live Biotherapeutic Products
I.	*Donor Screening*	**++**	**+++**
II.	*Sample Screening*	**?**	**+++**
III.	*Product Manufacturing* *Procedure*	**?**	**+++**
IV.	*Clinical Trial Data*	**+**	**+++**
V.	*Safety Data*	**+**	**+++**
VI.	*Ease of Access*	**?**	**+**

+ Good.

++ Better.

+++ Best.

? Unknown/Not applicable.

## 3 Fecal microbiota transplantation versus live biotherapeutic products

### 3.1 Donor screening

Screening of potential donors for FMT has evolved from local screening and rudimentary methods of stool processing in an endoscopy suite prior to a colonoscopic application of the blended material to more rigorous screening and packaging of the samples with the development of stool banks. For FMT, the screening process usually includes a rigorous history to exclude chronic usage of medications that can impact the microbiota, chronic diseases thought to be associated with dysbiosis of the microbiota, such as diabetes mellitus, metabolic syndrome, and a variety of blood tests including testing for human immunodeficiency virus, hepatitis A, hepatitis B and hepatitis C and stool testing for CDI, ova and parasites (Bakken et al., 2011). For FMT, there are no “standard” screening criteria for the donor and it is recommended that screening be comprehensive (Peery et al., 2024). Donor screening criteria are not uniform across individual institutions or stool banks and is site dependent (Tariq et al., 2018). Stool banks require FDA oversight under an investigational new drug (IND) application, therefore, meeting a standardized minimum requirement that is not necessarily met by local sites. Donor screening for LBPs has undergone rigorous assessment by the FDA having comprehensive safety checks to ensure consistent expansive safety within each sample distributed.

### 3.2 Sample screening

FMT procedures typically require comprehensive donor screening, but once the sample is donated, there is no further assessment of the microbial contents of what will be administered to the recipient. It is assumed that since this is a broad spectrum of microorganisms that sufficient levels of *Bacteroidetes* and *Firmicutes* are being administered, in the case of CDI, but the volume and proportion of these bacterial phyla, or any other microorganisms is not regularly assessed. In contrast, once the sample has been obtained from donors for LBPs, there is a quality assurance that what is administered is consistent with the proprietary formulation that was used in the FDA overseen clinical trials. Thus, LBPs have more consistent end-products, resulting in more predictable safety and efficacy.

### 3.3 Product manufacturing procedure

The manufacturing process is very heterogenous for individual institutions that are not engaging with a stool bank for the samples they will administer. There was no codified format for production of these samples and each institution mixed the samples differently dependent upon resources available to the clinical care team. Moreover, the process of administration, be it through colonoscopy or oral administration, added an extra layer of heterogeneity in its use. Stool banks are more regulated, and follow good clinical practice for their production under their IND application with the FDA (FDA, 2013). The LBPs are approved by the FDA, and as a result, always are produced under Good Manufacturing Practice, having regulation and uniform manufacturing procedures that are approved by FDA.

### 3.4 Clinical trial data

Most trial data for FMT varies in terms of the tools used for diagnoses, the time measured to recurrence, FMT preparation techniques, modes of administration, criteria for an adverse event and outcomes assessed - all of which have led to significant heterogeneity of this data. Trial data pertaining to LBPs is more uniform by virtue of its FDA trial oversight, standardized inclusion criteria, processing techniques and formulation as well as administration, making the data more codified, reliable and reproducible.

### 3.5 Safety data

Adverse events following FMT are common, but are mostly mild and include abdominal discomfort, diarrhea, nausea, vomiting and constipation (Saha et al., 2021; Kelly et al., 2021b). Serious adverse events have been seen in <10% patients and the majority are thought to be unrelated to FMT (Saha et al., 2021; Kelly et al., 2021b). A small number of bloodstream infections caused by organisms in FMT have been documented as well (Solari et al., 2014; DeFilipp et al., 2019), however most of such cases have occurred in patients who are immunocompromised and therefore at a high risk of bacterial translocation. In contrast, safety data extracted from LBP trials have revealed a similar frequency of events with the majority being mild to moderate adverse events of similar gastrointestinal nature and thus far there are no reports of infectious diseases being transmitted by LBPs (Lee et al., 2023). Further, controlled clinical trials require more stringent safety reporting, including solicited adverse events and adjudication by independent Drug and Safety Monitoring Boards.

### 3.6 Ease of access

The last, but perhaps most clinically relevant factor is ease of access of both of these therapies. As stated above, FMT cannot be undertaken by a physician or a practice without an IND through the FDA or via a stool bank with an approved IND with the FDA. While LBPs, being approved by the FDA, can be administered by any licensed and practicing physician in the appropriate clinical setting. The question of cost and insurance coverage is one that is still being defined.

With this foundational knowledge in mind, let’s discuss these two ground-breaking new LBP therapies in more detail.

## 4 Fecal microbiota live-JSLM (Rebyota™, RBL)

RBL was the first FDA approved LBP consisting of a broad consortium of spore and non-spore forming bacteria, including a minimum threshold of *Bacteroides*. It is a single, rectally administered dose containing 150mL of therapeutic material and 10^7^ microbes per mL or 15 x 10^8^ microbes per treatment.

### 4.1 Clinical trial data

A phase III prospective, randomized, double-blind, placebo-controlled trial (PUNCH CD3) assessed the efficacy of RBL in patients with the one or more recurrences (Khanna et al., 2022a). Diagnosis for entry into the study was at the discretion of the local investigator including PCR and EIA/glutamate dehydrogenase (GDH). Patients were treated with SOC antibiotics for a minimum of ten days, followed by a washout period of one to two days, after which they were randomized to either receive a single dose of RBL or a single dose of placebo via rectal instillation. Following 8-weeks, the overall model adjusted efficacy of the SOC+RBL was 70.6% compared with 57.5% for the SOC+placebo. A Bayesian statistical analysis leveraging data from the phase II randomized controlled trial was utilized, demonstrating a posterior probability of superiority of 0.991 and statistical significance. The PUNCH CD3 open label study, was reported as an interim analysis assessing 300 patients with first recurrence and beyond who had medical co-morbidities that were not included in the original phase 3 trial including irritable bowel syndrome and inflammatory bowel disease. Of these patients, 74.6% had a clinical resolution at 8 weeks after treatment with RBL; of those 84.0% remained responsive 6 months after treatment (Khanna et al., 2022b). There were no concerning adverse or serious adverse events observed in either trial.

### 4.2 Constitution of fecal microbial structure and resultant metabolome

To further prove the durability of these results with the clinical success demonstrated by this trial, the fecal microbial constitution of patients was investigated. Patients submitted stool samples prior to treatment, and then at 1, 4 and 8 weeks, 3 and 6 months after receiving study treatment (Blount et al., 2021). The microbiome of patients who received RBL showed a rapid shift with an increase in favorable *Bacteroides* and *Clostridia* and decrease in the proinflammatory *Gammaproteobacteria* and *Bacilli*. Furthermore, this change was durable during the 6 month follow up.

Bile acids are known to play a critical role in the life cycle of *Clostridioides difficile.* Primary bile acids trigger the spore germination and permit growth of *C. difficile* while secondary bile acids inhibit this process and interfere with the propagation of the infection (Winston and Theriot, 2016). Further testing the credibility of RBL, the stool samples collected from participants were also evaluated for changes in bile acid compositions (Papazyan et al., 2021). Here, they found that primary bile acids predominated before treatment, while secondary bile acids were more prevalent after treatment, and these changes were more significant in the patient population treated with RBL, further validating its downstream biochemical effects.

### 4.3 Administration

The product is administered 24-72 hours after completion of the SOC antimicrobial course for CDI. The application kit consists of a broad microbial consortium in 150mL of material contained within a small plastic bag, tubing and a clamp on the tube. Prior to administration, the patient is appropriately positioned in a left lateral decubitus or knee to chest position (Feuerstadt et al., 2023). One end of the tube is inserted into the bag containing the microbial consortium while the other end is lubricated and placed into the patient’s rectum. The bag is slowly raised by the provider administering the treatment and the clamp attached to the tube is opened to allow free flow of the material through gravity. The material flows in over 2-3 minutes and the patient is then maintained on their side for 10 minutes for observation.

RBL is very different from traditional FMT for several reasons, most of which were outlined as part of the FMT versus LBP section. It is probably most important to understand that, although RBL is a broad consortium, there is sampling of the post-donation consortium to ensure consistency of safety and efficacy with what has been tested previously in clinical trials, including ensuring a minimum threshold of *Bacteroides*. A marked difference between this type of product and the heterogenous FMT, with no sampling prior to administration. The better structured trials and consistency of administered product should result in wider acceptance and application by clinicians.

## 5 Fecal microbiota spores, LIVE BRPR (Vowst™)

Vowst (VOS) is distinct from RBL as this is a microbiota-based LBP that is derived from donor stool and subjected to an ethanol purification process that results in the isolation of *Firmicutes* spores. This is a consortium of microorganisms that only produce spores. VOS is administered orally as four capsules a day for three consecutive days after the patient has completed a course of SOC antimicrobials and a bowel lavage.

### 5.1 Clinical trial data

A phase III, prospective, double blind, randomized, placebo-controlled trial (ECOSPOR III) included patients with two or more recurrent episodes of CDI diagnosed by enzyme immunoassay or cell cytotoxicity neutralization assay (Feuerstadt et al., 2022b). Patients enrolled received ten to twenty-one days of SOC, followed by a washout period, after which magnesium citrate was administered the day of, or the night before intervention. Patients were randomized to receive either VOS or placebo and were monitored for recurrence for 8 weeks. By the end of this 8-week time period, 88% patients who received SOC+VOS demonstrated sustained clinical response, as compared with 60% of those who received SOC+placebo (p<0.001). There were no concerning safety signals within this trial.

A subsequent phase III, prospective, single arm, open-label trial (ECOSPOR IV) investigated the safety of VOS through weeks 24 by expanding their inclusion criteria and formulating two separate cohorts of patients (Sims et al., 2023). They included patients from the ESCOPOR III trial who recurred as part of the “first” cohort, and the “second” cohort included those rCDI patients diagnosed via PCR assay and/or those with first recurrence of the infection. They found an overall efficacy of 91.3% at 8 weeks and 86.3% at 24 weeks. Those with first recurrence had a 93.5% efficacy at 8 weeks.

### 5.2 Constitution of fecal microbial structure and resultant metabolome

The fecal microbiota composition of enrolled patients was also investigated to detect the microbial changes resulting from the administration of VOS. Stool specimens were obtained at baseline, which was within three days of completion of SOC, and then at 1, 2 and 8 weeks. The composition of microbiota in patients who received SOC+VOS revealed a higher prevalence of *Firmicutes* including *Ruminococciae and Lachnospiriciae* and a decrease in the proinflammatory *Enterobacteriaceae*. This was evident by week 1 and persisted through week 8. This supported the evidence of engraftment of the product with a desirable microbial response. Further, the investigated metabolomic profile revealed that secondary bile acids were significantly more prevalent in the SOC+VOS arm compared with SOC+placebo at 1 week and 8 weeks, exhibiting an overall favorable downstream effect and providing a good metabolic explanation for the success of those treated with VOS.

### 5.3 Administration

VOS should be administered two to four days after completion of SOC antibiotics to ensure wash out of the SOC antimicrobial. The day prior to administration, or at least 8 hours before the first dose, patients should drink 10oz of magnesium citrate or 250 mL of polyethylene glycol (if with renal insufficiency). VOS is consumed orally, with a total of 4 capsules taken daily for 3 days on an empty stomach prior to the first meal of the day.

## 6 Important considerations for phase III trials of LBPs

With an understanding of the clinical trials for RBL and VOS, it is also important to consider some key factors that might impact these trials, and how these factors might affect outcomes measured. It is pertinent to note that in each trial, both the intervention arm and the placebo arm received SOC antibiotics prior to randomization. The LBP was an “add-on” to the standard of care to improve its efficacy. The “placebo” arm frequently implies that patients did not receive further active intervention following SOC antibiotics. The duration of antimicrobials did differ among the trials ranging from ten to twenty-one days in the VOS trial and a minimum of 10 days in the RBL. It is unclear how this might impact outcomes, but longer courses of antimicrobials, such as vancomycin, might not be better, given the impact of vancomycin on the microbiota (Vrieze et al., 2014).

When considering treatment of a disease, if the disease is not properly diagnosed, one can’t expect a treatment targeting that disease to work. Therefore, diagnostic testing plays an essential role. Unfortunately, the diagnostic tools we have for CDI are sub-optimal. The polymerase chain reaction (PCR) assay, detecting the genes that code for the toxin, is the most commonly used assay for CDI in the United States but has a tendency to over-diagnose the infection, being positive when the *C. difficile* is not necessarily releasing toxin (Magill et al., 2018). Alternatively the enzyme immunoassay (EIA), a test that detects the toxin itself, has an unacceptably low sensitivity (Khanna et al., 2017). Therefore, a negative test does not necessarily rule out CDI. Finally, a cell culture cytotoxin neutralization assay is an accurate tool for diagnosis, but not widely available in clinical practice. Testing in the clinical trials need to balance accuracy while also mimicking what is available for the majority of practitioners. Ultimately, the diagnostics used in the trial have an impact on accurate patient selection and therefore the ability of interventions to show they work in the correct patients.

The number of recurrent episodes used for initial enrolment was not uniform among the LBP trials. The pivotal trial for RBL included patients with one or more recurrent episodes while the VOS trial included patients with two or more rCDI episodes. It is believed that with each recurrence, the dysbiotic state of the patient becomes more severe. Therefore, if we are trying to use a therapy that restores the deficiencies, one might argue that with more recurrences, it might require more therapy to achieve the same effect in getting the microbiota above the threshold where it can effectively resist germination of *C. difficile* and a recurrence.

A washout period is the time between completion of antimicrobials and administration of the LBP. The purpose of the washout period is to ensure that the SOC antibiotics have been eliminated from the patient so they do not significantly alter the newly administered organisms given within the LBPs. This washout period ranges between 24 and 72 hours, which could further be confounded by the specific antimicrobial therapy that was received prior to LBP.

Further, only the VOS trial used a bowel purge to further eliminate the SOC antimicrobial from the patient. This was performed with magnesium citrate. Dosing of each LBP also varies significantly. While RBL is a 150mL enema given once and is a broad consortium of microorganisms including a minimum threshold of *Bacteroidetes*, VOS is administered in the form of capsules over 3 days and is a narrow consortium of *Firmicutes* microorganisms.

## 7 Discussion

For years, treatment avenues for CDI have been limited to SOC antimicrobial therapies, including vancomycin and fidaxomicin. While these antibiotics are effective and are supported by current clinical guidelines (Johnson et al., 2021; Kelly et al., 2021a), they only treat a portion of the infection and can further contribute to the disruption of the gut microbial environment. They also cannot act effectively on the *C. difficile* spores, which can remain in a patient’s system and if the microbiota is unable to properly regrow and rediversify, these spores can germinate causing a recurrence.

With the discovery of the pivotal role that the intestinal microbiota plays in the pathogenesis of CDI, recent therapeutics sought to modulate the microbiome and its resultant metabolome hindering recurrence of CDI. Fecal microbiota transplantation was the first microbial remodulation therapy to be developed. While evidence for FMT was reassuring, the lack of standardization across studies raised concerns with its safety along with risks of transmission of other pathogenic organisms (DeFilipp et al., 2019). With the need for more uniform therapies, that a wider array of providers would feel comfortable using, the LBPs were developed, which were created in a more regulated fashion having much more rigidly structured clinical trials testing their safety and efficacy. Clinical trial data for both RBL and VOS was encouraging, proving not only their clinical success, but also their sustained efficacy at a microbial and metabolomic level ultimately resulting in FDA approval of both for the prevention of recurrence of CDI. With this approval, there should be much wider access for these effective therapies for both providers and patients. As the frequency of their administration increases in clinical practice, both products should result in a significant reduction in the rates of rCDI and hopefully the burden of CDI on our patients and healthcare system.

## Author contributions

KS: Conceptualization, Data curation, Writing – original draft. PF: Conceptualization, Supervision, Writing – review & editing.
